# Environment-Sensitive Intelligent Self-Reproducing Artificial Cell with a Modification-Active Lipo-Deoxyribozyme

**DOI:** 10.3390/mi11060606

**Published:** 2020-06-22

**Authors:** Muneyuki Matsuo, Yuiko Hirata, Kensuke Kurihara, Taro Toyota, Toru Miura, Kentaro Suzuki, Tadashi Sugawara

**Affiliations:** 1 Department of Basic Science, Graduate School of Arts and Sciences, The University of Tokyo, Komaba, Meguro, Tokyo 153-8902, Japan; muneyuki@hiroshima-u.ac.jp (M.M.); cttoyota@mail.ecc.u-tokyo.ac.jp (T.T.); 2 Department of Creative Research, Exploratory Research Center on Life and Living Systems (ExCELLS), Myodaiji, Okazaki, Aichi 444-8787, Japan; kkurihara@jamstec.go.jp; 3 Department of Chemistry, Faculty of Science, Kanagawa University, Tsuchiya, Hiratsuka, Kanagawa 259-1293, Japan; yuikohirata@gmail.com (Y.H.); suzuken@kanagawa-u.ac.jp (K.S.); 4 Department of Life and Coordination-Complex Molecular Science, Biomolecular Functions, Institute for Molecular Science, Myodaiji, Okazaki, Aichi 444-8585, Japan; 5 Universal Biology Institute, The University of Tokyo, Komaba, Meguro, Tokyo 153-8902, Japan; 6 Misaki Marine Biological Station, School of Science, The University of Tokyo, 1024 Koajiro, Misaki, Miura, Kanagawa 238-0225, Japan; miu@mmbs.s.u-tokyo.ac.jp

**Keywords:** artificial cell, giant vesicle, self-reproduction, DNA amplification, lipo-deoxyribozyme, primitive information flow, phenotypic plasticity, competitive proliferation, intelligent supramolecular machine

## Abstract

As a supramolecular micromachine with information flow, a giant vesicle (GV)-based artificial cell that exhibits a linked proliferation between GV reproduction and internal DNA amplification has been explored in this study. The linked proliferation is controlled by a complex consisting of GV membrane-intruded DNA with acidic amphiphilic catalysts, working overall as a lipo-deoxyribozyme. Here, we investigated how a GV-based artificial cell containing this lipo-deoxyribozyme responds to diverse external and internal environments, changing its proliferative dynamics. We observed morphological changes (phenotypic expression) in GVs induced by the addition of membrane precursors with different intervals of addition (starvation periods). First, we focused on a new phenotype, the “multiple tubulated” form, which emerged after a long starvation period. Compared to other forms, the multiple tubulated form is characterized by a larger membrane surface with a heavily cationic charge. A second consideration is the effect of the chain length of encapsulated DNA on competitive proliferation. The competitive proliferation among three different species of artificial cells containing different lengths of DNA was investigated. The results clearly showed a distinct intervention in the proliferation dynamics of the artificial cells with each other. In this sense, our GV-based artificial cell can be regarded as an intelligent supramolecular machine responding to external and internal environments, providing a new concept for developing molecular machines and robotics.

## 1. Introduction

**Figure 1:**
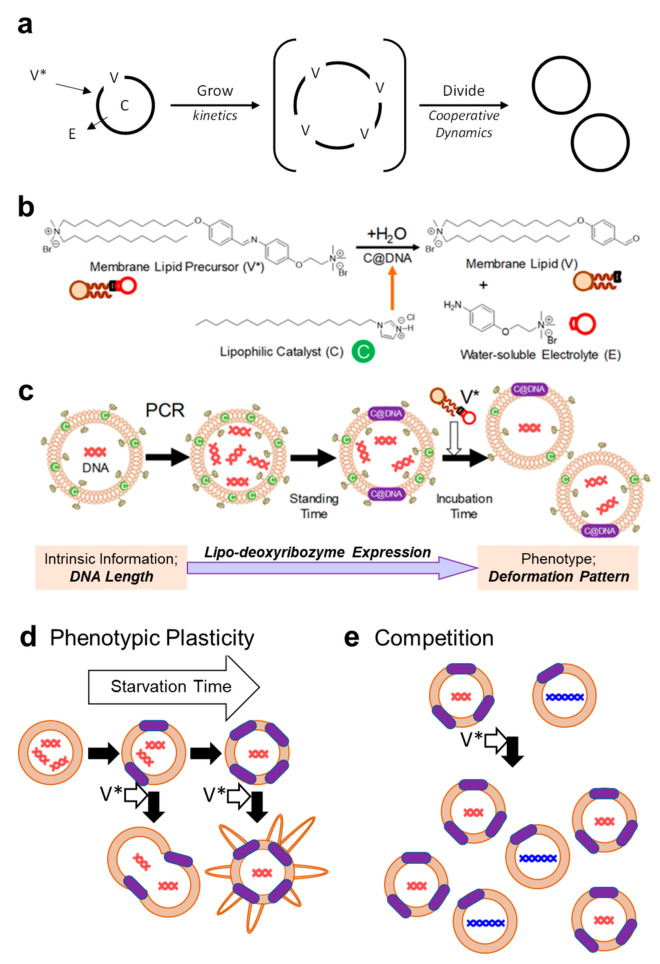
Self-reproduction of GV-based artificial cells working as self-reproducing molecular machines (**a**). Relevant molecular structures. V* is a precursor of membrane lipids (nutrients), **V** is a membrane lipid, E is a water-soluble electrolyte, and **C** is an amphiphilic catalyst (**b**). DNA amplification in GV-based artificial cells and the formation of **C**@DNA and its proliferation induced by the addition of V* (**c**). Artificial cells exhibiting phenotypic plasticity depending on starvation time (**d**). Competitive proliferation between GVs incorporating different DNA lengths. Red double helices represent short DNA and blue double ones represent long DNA (**e**).

Model artificial cells have drawn considerable attention from the viewpoint of revealing the origin of life and its intrinsic nature [1,2]. Since a compartment of an artificial cell is made of a giant vesicle (GV, diameter ≥ 1 μm) that exhibits semipermeability [3], water and small nonionic molecules are able to go in and out through the membrane. The best nonequilibrium dynamics of this artificial cell involve self-reproduction (Figure 1a). The macroscopic self-reproduction of the GV-based artificial cell must occur before the generation of membrane molecule **V** from its precursor V* is equilibrated. In fact, the forward reaction (generation of **V**) is an imine hydrolysis and it is in equilibrium with a backward reaction (consumption of **V** and **E** to form V*). A newly formed GV membrane serves as a reaction environment to proceed the formation of membrane molecule **V** again. A coupling of chemical generation of **V** from V* and macroscopic production of GV keeps the self-reproduction of GV to proceed repeatedly (Figure 1b) [4]. Namely, the GV-based artificial cell works as a self-reproducing molecular machine. However, the division frequency of this simple self-reproducing system was reported to be low.

To make a GV-based artificial cell more productive, it is inevitable to install a regulation system to induce GV division with a high frequency. In a previous study [5], we encapsulated polymerase chain reaction (PCR) reagents for DNA replication in GVs, such as template DNA, deoxyribonucleotides (dNTPs), DNA polymerase, and Mg^2+^, by a freeze–thaw technique. When the membrane precursor V* was added to a dispersion of GVs in which DNA was amplified, it was found that such GVs divided rapidly with high frequency (Figure 1c).

Careful investigations [6] revealed that the origin of this accelerated division of GV with amplified DNA came from a supramolecular complex of GV membrane-intruded DNA and cationic catalyst **C** (abbreviated as **C**@DNA), which catalyzed the conversion from V* to **V**. We found the emergence of a primitive information flow in our GV-based artificial cell, starting from the amplification of DNA, the accumulation of **C**@DNA in the GV membrane, the accelerated formation of membrane lipid **V** around **C**@DNA, and the induction of an equivolume division as a result of budding deformation. In this flow, DNA in **C**@DNA is an information molecule and works as a cocatalyst to convert V* to **V** in the GV membrane. Notably, in this primitive artificial cell, the information is not derived from the DNA sequence but from the DNA chain length. Consequently, **C**@DNA as a whole can be regarded as a lipo-deoxyribozyme, in reference to ribozymes. Thus, this self-proliferation system can be regarded as a lipo-deoxyribozyme-controlled supramolecular robot.

This information flow is still very flexible compared with a well-established transcription and translation system in a contemporary living cell [7], therefore it is possible to be modified by responding to the external environment, such as a starvation period, which corresponds to a time interval for encountering nutrients (Figure 1d). Under nutrient-poor conditions, a different phenotype, called multiple tubulated GV, emerged. The preying-on behavior of this new phenotype was investigated. We also studied the competitive proliferation between three different species of artificial cells containing different lengths of DNA (374, 1164, or 3200 bp) by measuring the proliferation frequencies of the species by means of confocal laser scanning microscopy and flow cytometry (Figure 1e). The results clearly showed the distinct intervention derived from severe competition for nutrition source V* between different types of artificial cells in the proliferation dynamics.

These environment-sensitive, GV-based artificial cells, which are derived from the modification of lipo-deoxyribozymes, are expected to provide a unique concept to supramolecular robots to react and behave spontaneously under diverse environments [8].

## 2. Materials and Methods

### 2.1. Materials

1-Palmitoyl-2-oleoyl-*sn*-glycero-3-phosphocholine (POPC), 1-palmitoyl-2-oleoyl-*sn*-glycero-3-phospho-(1-*rac*-glycerol) sodium salt (POPG), and 1,2-distearoyl-*sn*-glycero-3-phosphoethanolamine-*N*-(methoxy(polyethylene glycol)-1000) (DSPE-PEG1000) were purchased from Avanti Polar Lipids, Inc. (Alabaster, AL, USA). Cholesterol and 4-hydroxyl-TEMPO (TEMPOL) were purchased from Wako Pure Chemical Industries (Osaka, Japan). The membrane lipid molecule **V**, the amphiphilic catalyst **C**, the catalyst **C** tagged with 4,4-difluoro-4-bora-3a,4a-diaza-*s*-indacene (BODIPY) and the membrane lipid precursor V* were prepared as previously reported [4,5]. The restriction enzyme EcoRI, the pBR322 Vector Wizard^®^ SV gel and the PCR clean-up system for purification were purchased from Promega Corporation (Madison, WI, USA). Primers and a primer tagged with Texas Red were purchased from Sigma-Aldrich (St. Louis, MO, USA). DNA ladder markers and DNase I were purchased from Takara Bio Inc. (Shiga, Japan). KOD-Plus DNA polymerase was purchased from Toyobo Co., Ltd. (Osaka, Japan). SYBR^®^ Green I was purchased from Lonza (Rockland, ME, USA). Texas Red 1,2-dihexadecanoyl-*sn*-glycero-3-phosphoethanolamine, triethylammonium salt (Texas Red-DHPE), 2-(4,4-difluoro-5,7-dimethyl-4-bora-3a,4a-diaza-*s*-indacene-3-dodecanoyl)-1-hexadecanoyl-*sn*-glycero-3-phosphocholine (BODIPY-HPC), flow cytometry size calibration kit (nonfluorescent microspheres) and SYBR^®^ Gold nucleic acid gel stain were purchased from Thermo Fisher Scientific Inc. (Waltham, MA, USA). TE-saturated phenol:chloroform:isoamyl alcohol (25:24:1, v/v/v) was purchased from Nacalai Tesque, Inc. (Kyoto, Japan). The coprecipitating agent ethachinmate and sodium acetate (3 M, pH 5.2) for ethanol precipitation were purchased from Nippon Gene Co., Ltd. (Tokyo, Japan). Precast 12.5% poly(acrylamide)gel (e-PAGEL minigel, AE-6000 E-T12.5 L) was purchased from Atto Corporation (Tokyo, Japan).

### 2.2. General Protocol for Preparation of Giant Vesicle (GV)-Based Artificial Cells

Similar to our previous investigations [5,6], a preparation of GV-based artificial cells, DNA excision, DNA amplification and polyacrylamide gel electrophoresis were conducted. Membrane lipid compositions of phenotypic plasticity and competitive proliferation experiments were POPC:POPG:**V**:**C**:DSPE-PEG1000:cholesterol = 35:39:12:9:0:5 and 78:0:4.2:8.5:8.5:0.85, respectively. GVs containing PCR reagents were prepared by the freeze–thaw method as follows. Stock methanol solutions of the lipids were prepared independently and were mixed in a test tube. The mixture was dried to a thin lipid film under reduced pressure to remove the solvent. The dry lipid film was hydrated by addition of the deionized water, followed by vortex mixing for 30 s, resulting in the formation of a GV dispersion. The GV dispersion was incubated for 2 h at 25 °C. It was then rapidly frozen in liquid nitrogen and stored under reduced pressure over a period of 4.5 h. The resulting lipid powder was rehydrated with 500 µL of a PCR solution, deionized water (347 µL), PCR buffer (10 × KOD-plus-buffer, 50 µL), MgSO_4_ aq. (25 mM, 20 µL), dNTPs mixture solution (each 2 mM, 40 µL), forward primer (10 µM, 14 µL), reverse primer (10 µM, 14 µL), template DNA (10 nM, 5 µL), DNA polymerase (0.6 μM, 1.0 U/μL KOD-Plus-, Mg^2+^ free, 10 µL). In order to stabilize the resulting GVs, the dispersion was incubated for at least 1 h at 25 °C. After the incubation, in order to digest DNA of exterior of the GVs, the buffer solution (500 µL) containing DNase I was added to the dispersion. The contents of outer buffer solution were as follows; deionized water (435 µL), PCR buffer (10 × KOD -plus-buffer, 50 µL), CaCl_2_ aq. (100 mM, 5 µL), DNase I (5,000 U/µL, 10 µL). The solution was incubated for 30 min at 25 °C.

### 2.3. Microscopy Observation for the Deformation Pattern of GV-Based Artificial Cells

A 2.5 mM solution of V* was prepared by dissolving V* in a mixture of deionized water (445 µL), PCR buffer (10 × KOD-Plus-buffer, 50 µL) and CaCl_2_ aq. (100 mM, 5 µL). The resulting V* solution was ultra-sonicated for 10 min. Then, 10 µL of the V* solution was immediately added to 10 µL of the prepared dispersion of GV (lipids concentration; 2.5 mM), after the starvation period of, e.g., 3 h. Then, the mixture was placed in a glass plate with a cover glass under a differential interference contrast (DIC) microscope (BX51, OLYMPUS, Tokyo, Japan). Microscopy images for capturing the dynamics of GVs in the dispersion were recorded by using a DIC microscope equipped with a Charge Coupled Device (CCD) camera (DP30BW, OLYMPUS, Tokyo, Japan).

### 2.4. Preparation of GVs for Förster-Type Energy Transfer (FRET) Experiments

To prepare GVs containing 1164 bp-DNA tagged with Texas Red, the freeze-dried lipid powder composed of POPC:POPG:**V**:**C**:**C**-BODIPY:cholesterol (35:39:12:8:1:5, mol%) were hydrated with 500 µL of a PCR solution composed of deionized water (115 µL), PCR buffer (10 × KOD-Plus buffer, 50 µL), MgSO_4_ aq. (25 mM, 20 µL), dNTP mixture solution (2 mM each, 250 µL), forward primer-Texas Red (100 µM, 15 µL), reverse primer (100 µM, 15 µL), 1164 bp-DNA template (10 nM, 25 µL), and DNA polymerase (1.0 U/μL, KOD-Plus, Mg^2+^ free, 10 µL). A dispersion of GVs were incubated at 25 °C for 1 h after the addition of DNase I, and the resulting GV dispersion was subjected to thermal cycles for the amplification of template DNA.

### 2.5. FRET from the Catalyst C Tagged with BODIPY to DNA Tagged with Texas Red-DHPE.

As reported by our previous report on the similar experiment [6], FRET from a donor (**C**-BODIPY) to an acceptor (DNA-Texas Red) was measured by a confocal microscope (LSM 5 LIVE, Zeiss, Tokyo, Japan) using three separate channels: a donor channel (**C**-BODIPY, 488 nm excitation/505–530 nm emission), an acceptor channel (DNA-Texas Red, 532 nm excitation/560–585 nm emission) and a FRET channel (488 nm excitation/560–585 nm emission) under the same gain, exposure time and laser intensity. The fluorescence intensity from individual target probes in the GV membrane was evaluated from the averages of the maximum intensities of 8-line profiles crossing over the GVs.

### 2.6. Microscopy Observation for Interaction between PreBD and MT

GV dispersions were prepared in a manner of “General protocol for preparation of GV-based artificial cells” as mentioned above. A GV dispersion with a 12-h starvation time after the PCR was named pre-budding (BD). Other GV dispersions with a 48-h starvation time after PCR were named pre-multiple tubulation (MT). At 12 h, after the prepared dispersion of GV (lipids concentration; 2.5 mM) were mixed to an equivolume of the V* solution (2.5 mM), pre-BD and pre-MT were renamed as BD and MT, respectively. Dispersions (13 μL) of different GVs were placed in a frame chamber (9 × 9 mm, 25 μL) on a glass plate, and then mixed under a confocal laser scanning (CLS) microscope. GVs of pre-BD/BD and pre-MT/MT were stained with Texas Red-DHPE (0.1 mol%) and BODIPY-HPC (0.1 mol%), respectively. Microscopy images for capturing the GVs in the mixture dispersion were recorded by using an inverted microscope (Eclipse Ti-E, Nikon Instruments Inc., Tokyo, Japan) equipped with a confocal laser scanner unit (CSU-W1, Yokogawa Electric Corp., Tokyo, Japan) and a sCMOS camera unit (Zyla 4.2 plus, Andor Technology Ltd., Belfast, United Kingdom) with 1024 × 1024 active pixels in two channels: a BODIPY channel (488 nm excitation/500–550 nm emission) and a Texas Red channel (561 nm excitation/544–690 nm emission).

### 2.7. Population Tracing of Mixture Dispersion of Pre-BD and MT

Population analysis of mixture dispersion of pre-BD stained with Texas Red DHPE and MT stained with BODIPY-HPC was conducted using a fluorescence-activated flow cytometer (FACS) (SH800, SONY, Tokyo, Japan) with two channels: a BODIPY channel (488 nm excitation/500−550 nm emission) and a Texas Red channel (561 nm excitation/570–600 nm emission). A dispersion of pre-BD was mixed with a MT dispersion at ratios of 1 to 3, 1 to 1 and 3 to 1, keeping the total volume of mixture at 500 μL. The original and mixture solutions were collected and then measured by FACS.

### 2.8. Counting Increased Numbers of GVs by CLS Microscopy

A dispersion (lipids concentration; 1 mM) of GVs that were stained by BODIPY-HPC (0.1 mol%) or Texas Red-DHPE (0.1 mol%) was prepared by freeze-drying, and the DNA was amplified by PCR. After a 48-h starvation period, the different dispersions of GVs stained with different fluorescence probe were mixed with a membrane precursor V* solution (1 mM) at a volume ratio of 1:1. Then, the mixture was immediately placed into a frame chamber (9 × 9 mm, 25 μL) on a glass plate with a cover glass. The GVs in five 1350 × 1350 μm x–y observation fields (four corners and one in the middle) in the frame chamber were captured as an x–y-sliced image using an inverted microscope (Eclipse Ti, Nikon, Tokyo, Japan) equipped with a confocal laser scanner unit (CSU-W1, Yokogawa Electric Corp., Tokyo, Japan) and an sCMOS camera unit (Zyla 4.2 plus, Andor Technology Ltd., Belfast, United Kingdom) with 2048 × 2048 active pixels in two channels: a BODIPY channel (488 nm excitation/500–550 nm emission) and a Texas Red channel (561 nm excitation/544–690 nm emission). Along the z axis, 25 x–y-sliced images were captured with a z-interval of 10 μm to constitute a z-stack. All GVs on 125 sliced images (25 images × 5 x–y positions) were counted by viewer and analyzer software (NIS-Elements, Nikon, Tokyo, Japan). The percent increase was calculated from the counted numbers of GVs before and after the addition of V*.

### 2.9. Counting Increased Numbers of GVs by Highly Sensitive CLS Microscopy

Dispersions of GV(M) (lipids concentration; 1.3 mM) stained with BODIPY-HPC (0.1 mol%) and GV(S) (lipids concentration; 1.3 mM) stained with Texas Red-DHPE (0.05 mol%) were prepared separately by freeze-drying, and DNA in each GV was amplified by PCR. After a 72-h starvation period, both dispersions of GVs stained with different fluorescence probe were mixed with a membrane precursor V* solution (1.3 mM) at a volume ratio of 1:1. Then, the mixture was immediately placed into a frame chamber (9 mm × 9 mm, 25 μL) on a glass plate with a cover glass. The GVs in five x–y observation fields with each area of 185 × 185 μm (four corners and one in the middle) in the frame chamber were captured as an x–y-sliced image using a confocal scanning laser microscopy (TCS SP8, Leica Microsystems, Wetzlar, Germany) with 512 × 512 active pixels through two channels: a BODIPY channel (488 nm excitation/505–540 nm emission) and a Texas Red channel (555 nm excitation/615–750 nm emission). Along the z axis, images were captured with a z-interval of 5 μm, constituting a z-stack. All GVs on images were counted by viewer (LAS X, Leica Microsystems, Wetzlar, Germany) and analyzer software (Fiji, National Institutes of Health, Bethesda, MD, USA).

### 2.10. Population Tracing of Competitive Proliferation between GV(M) and GV(S)

GVs were prepared in the same method as 2.9. GV(M) were stained by BODIPY-HPC (0.1 mol%) and GV(S) were stained by Texas Red-DHPE (0.1 mol%). After a starvation period of 72 h, both GV dispersions (1.3 mM) were mixed with an equivolume of V* dispersion (0.14 mM, 0.42 mM, 1.3 mM, 3.8 mM, or 11 mM) in the same sample tube. The prepared samples were set and measured by FACS (MA900, SONY, Tokyo, Japan) with two channels: a BODIPY channel (488 nm excitation/518 nm emission) and a Texas Red channel (488 nm excitation/615 nm emission), sequentially. The measurement was conducted at 10, 20, 30, 60, and 120 min after the addition of V*.

## 3. Results

The investigation was focused on the response of our self-reproductive, GV-based artificial cell towards severe external conditions; one is starvation of nutrients and the other is scrambling for the nutrients between artificial cells containing different DNA. The adaptative or competitive response of the GV-based artificial cell was derived from the difference in the concentration or structure of **C**@DNA which worked as lipo-deoxyribozyme in the GV membranes.

In Section 3.1, the appearance of a novel phenotype, a multiple tubulation, induced by the addition of V* to a PCR-subjected, GV-based artificial cell after a prolonged starvation time, e.g., 15 h was discovered, and the aggressive behavior of this phenotype was discussed. In Section 3.2, increased ratios of artificial cells containing different lengths DNA (L, M and S) under competing proliferation conditions were evaluated, and the appearance of the predominant species among them was recognized. These two distinct experimental results indicate that GV-based artificial cells have reached to the stage of a “living cell” at least from the aspect of the flexible response to the change of the external environments.

### 3.1. Phenotypic Plasticity

Living organisms can develop phenotypic diversities to adapt to a given environment, which is recognized as phenotypic plasticity [9,10]. Phenotypic diversity would have occurred more frequently in primitive cells, where the information flow was not as precise and robust as in today’s organisms, and would have had a greater influence on their evolution [11]. However, how primitive phenotypic plasticity was expressed in a prebiotic era has not been demonstrated experimentally. Hence, we investigated the emergence of the phenotypic plasticity of an artificial cell against a starvation condition, which was the environmental factor influencing its existence.

#### 3.1.1. Starvation Periods to Form Pre-Budding and Pre-Multiple Tubulated Phenotypes

**Figure 2:**
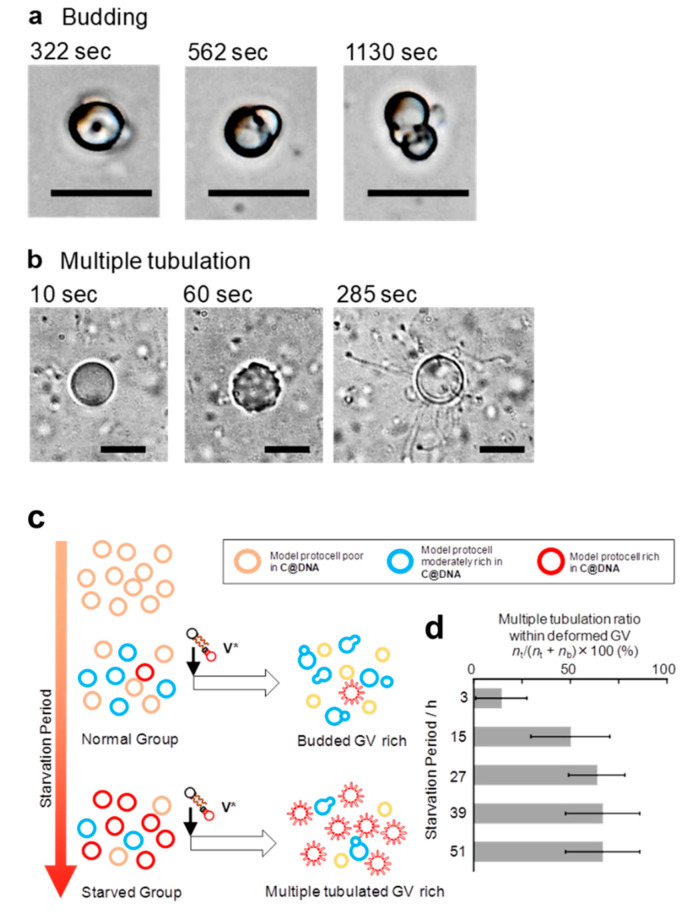
Two patterns of morphological changes observed by the addition of V* after starvation periods: DIC microscopy images of the budding (**a**), the time-lapse morphological changes to the multiple tubulation (**b**), followed by addition of membrane precursor V*. Scale bars represent 10 µm. Schematic illustration of the starvation period-dependent crossover from a normal group to a starved group (**c**), dependence of ratios of multiple tubulation to the whole morphologically changed GVs on the starvation periods. The ratio of multiple tubulation to total changes (multiple tubulation + budding) increased from 14% (2/14 protocells) after 3 h to 50% (3/6) after 15 h, and eventually saturated at ca. 65% (7/11), (4/6), (4/6) after 27, 39, and 51 h, respectively. The total number of GVs was obtained through 10 experiments. Error bars represent the confidence interval of 95% on the basis of the two-term distribution (**d**).

Other than the budding-type deformation (BD, Figure 2a), which is a regular deformation pattern (i.e., phenotype) in the reproduction dynamics, we found a novel phenotype, multiple tubulation (MT, Figure 2b), which was induced by the addition of a membrane precursor V* after a relatively long starvation time when we used GV-encapsulating, 1164-bp DNA with a membrane composition of 1-palmitoyl-2-oleoyl-*sn*-glycero-3-phosphocholine (POPC):1-palmitoyl-2-oleoyl-*sn*-glycero-3-phosphoglycerol (POPG):**V**:**C**:cholesterol = 35:39:12:9.0:5.0. It was found that the phenotype MT was obtained when the ratio of anionic POPG in the membrane composition was in the range of 24-39 mol%. The requirement for a relatively rich composition ratio of POPG was presumably due to appropriate electrostatic interactions between the negative surface charge of the GVs and the cationic bolaamphiphilic V*. The dependence of the composition of the negatively charged lipid POPG was examined, and the precise data are shown in Table S1.

The MT deformation occurred in a manner such that tubules grew omnidirectionally from the GV surface (Movie S1). Using thin lamellar artificial cells (diameter = approx. 10 μm) with the above membrane composition and 1164-bp DNA, we examined the dependence of the morphological changes on the duration of the starvation period (3, 15, 27, 39, and 51 h). It was observed that the starvation period-dependent transformation gradually proceeded from a BD- to an MT-dominant phase (Figure 2c). The ratio of MT to the total number of deformed artificial cells (MT + BD) increased from 14% after 3 h to 50% after 15 h and eventually saturated at approx. 65% after 27 h (Figure 2d, figures in parentheses are the counted MT number over the total specimen). This result suggests that the artificial cell perceives the duration of the starvation period as the external environment. The MT- and BD-type artificial cells can be recognized as different phenotypes because the chain length of the encapsulated DNA (1164 bp), which controls the self-reproduction dynamics as the internal information [6], was fixed, and only the timing to encounter nutrients was prolonged in a nutrient-poor environment.

In our GV-based artificial cell, it was postulated that MT formation was promoted by accumulated lipo-deoxyribozyme in a GV membrane during the prolonged starvation period (Note S1). Hence, we studied the dependence of the ratio of MT formation on the accumulation of lipo-deoxyribozyme in the GV membrane. The gradual formation of lipo-deoxyribozyme in the GV membrane during the starvation period was strongly suggested by the increased fluorescence intensity due to Förster-type energy transfer from the cationic catalyst **C** tagged with BODIPY-HPC to DNA tagged with Texas Red-DHPE during the time interval in the presence of a water-soluble quencher (Figure S1).

To reveal the influence of the accumulation of lipo-deoxyribozyme in the GV membrane on MT formation, we changed the amount of V* added and studied the variation in the formation ratio of MT to the total changes. The ratio of MT increases proportionally to the increase in the concentration of V* (Figure S2). It was concluded that the generation of the environment-dependent phenotype, MT, was rationalized by the accumulation of the lipo-deoxyribozyme in GVs due to the prolonged starvation time as a result of phenotypic plasticity (Note S1 and Figure S3).

#### 3.1.2. Interactive Dynamics between Phenotypes

To clarify what kind of active role these phenotypes bring to a primitive protocell, we arranged MT and BD that were incubated for 6 h after the addition of V* as well as GVs prior to the addition of V*, which were named pre-MT and pre-BD, respectively. Compared to those of pre-MT and pre-BD, the surface charges of MD and BD became more positive due to the cationic **V** formed in the GV membrane after the addition of V*. Two kinds of GVs in the different stages were mixed together to observe the interaction between these GV groups by confocal scanning laser microscopy and flow cytometry.

**Figure 3:**
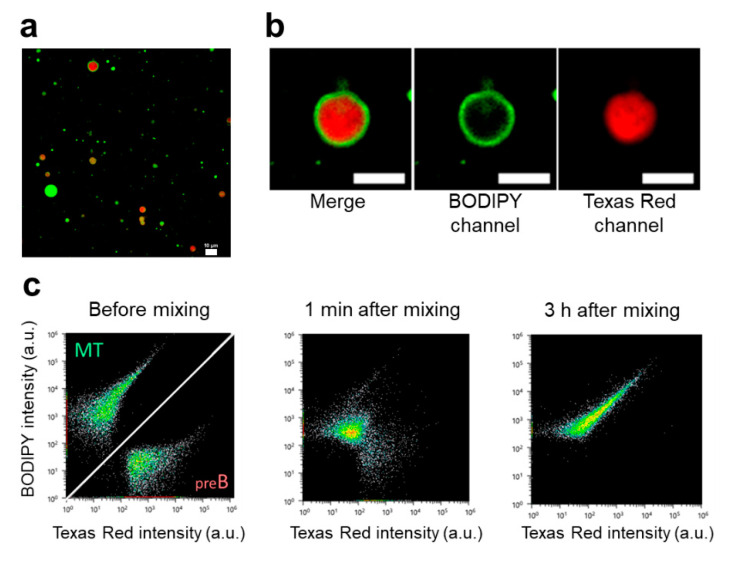
Intervesicular dynamics regarding coating pre-BD by MT. Confocal microscopy image of a mixed dispersion of MT tagged with BODIPY-HPC and pre-BD tagged with Texas Red-DHPE at 6 h after mixing (**a**), Confocal microscopy images of coated pre-BD with MT merged (channel), BODIPY channel, Texas Red channel, Scale bar represents 10 μm. (**b**, left, middle, right), Flow cytometry image of MT and pre-BD before addition of V*, image at 1 min after mixing, image at 3 h after mixing (**c**, left, middle, left). 3c left is a combination of two independent images separated by a diagonal white line. The above left image is assigned to the image of MT and the right below to the image of pre-BD before addition of V*.

Confocal microscopy images of a mixed dispersion of MT tagged with BODIPY-HPC and pre-BD tagged with Texas Red-DHPE were recorded at 6 h after mixing (Figure 3a). The image of Figure 3b was a representative expanded scope of Figure 3a showing a mixture of pre-BD and MT in the dispersion. The important result here was that almost all of mixed GVs in Figure 3b, originated from pre-BD and MT, emitting both red and green fluorescence light, although the distribution of mixed GVs was diverse in reference to the shapes of spherical GV, the degree of coating by MT, the degree of merging of two components (pre-BD and MT), and clustering between these two GVs (Figure S4). On the other hand, among groups of GVs prior to the addition of V* (pre-BD and pre-MT), no fusion between these groups was detected (Figure S5).

Hence, we measured ensemble of GVs (a mixture of pre-BD and MT) by flow cytometry to reveal population dynamics of GVs in the mixture. The data obtained by flow cytometry reflected the characteristics of enough amount of GV ensemble because the mas-scale data obtained by flow cytometry on 10,000 samples of GVs were shown in Figure 3c and Figure S6. The plots of each specimen exhibited positive correlation between BODIPY and Texas Red intensities in Figure 3c, strongly suggesting that predominant GVs contained the two components, pre-BD and MT, and the relative ratio of components preserved almost the same, regardless of the size of fused GVs. Before the addition of V*, the populations of MT and pre-BD were separate (Figure 3c, left). The image obtained at 1 min after the addition of MT to the dispersion of pre-BD showed that the populations of pre-BD and MT were fused instantly (Figure 3c, middle), and most of the pre-BDs in the dispersion were fused with MT at 3 h after mixing (Figure 3c right). The diagonally extended population suggests the clustering of two kinds of GVs. It is worth noting that no pre-BD (stained with BODIPY-HPC) fused with BD (stained with Texas Red-DHPE) was observed after pre-BD was mixed with BD (Figure S6). Based on the above microscopy images and the population analysis of dispersions with equivalent (Figure 3) or different mixing ratios (Figure S7), it is indicated that the pre-BDs were coated by multiple tubes originated from MTs.

The fluorescently heterogeneous GVs (and/or homogeneous GVs fluorescing in both green and red) observed under the fluorescence microscopy are plausibly formed by both pre-BD and MT. It is known that small vesicles can be layered on micrometer-sized particles due to the intermolecular attraction between the vesicular and the particle surfaces [12,13]. We thus interpret that, since MT were kinetically formed by addition of V* and the net charge of multiple tubules of MT was positive, the multiple tubules ruptured in presence of pre-BD the net charge of which was negative, and as a result, the vesicular fragments of MTs were entirely adhered around the surface of pre-BD.

The confocal microscopy and flow cytometry measurements revealed that the subtle difference in the surface charges of GVs extended to the interactive dynamics between groups of different kinds of GVs. Namely, the generated phenotype indicated the hierarchical interaction between groups of GV-based artificial cells.

### 3.2. Competitive Proliferation

In the preceding section, phenotype plasticity was discussed on the basis of flexible primitive information flow in response to the external environment. A question here is how the expression of phenotype is influenced if the DNA in GV-based artificial cells is varied. To answer this question, we prepared three types of GV-based artificial cells containing DNA with different chain lengths (3200, 1164, and 374 bp). The intrinsic proliferative ability of these artificial cells was estimated by noncompetitive conditions, and the distinct difference in the increase ratios between three types of artificial cells depending on DNA length, not on base pair sequence, has been revealed [6]. The increase ratio of GV(X)s (X = S, M, L) was defined as: Increase ratio (%) = [(2 × Number of GV(X)s after the addition V*/Number of GV(X)s before the addition V*) − 1] × 100. However, in nature, various species are competing, and a predominant species appears after natural selection [14].

Thus, it is significant to study the proliferation of GV (1164 bp) in the presence of other species of GV-based artificial cell, GV (3200 bp) or GV (374 bp) in the competitive environment. We expected that the difference in the increase ratios between GV (L), GV (M), and GV (S) must be enhanced if the proliferation ratio was measured in the competitive condition. However, the order of the increase ratios between GV (M) and GV (S) became smaller in the competitive proliferation due to the severe competition of scrambling for V*. It turned out that the concentration of V* affected the increase ratio significantly.

#### 3.2.1. Microscopy Observation of Competitive Proliferation

**Figure 4:**
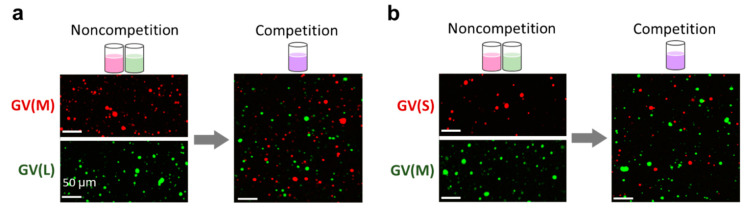
Competitive proliferation of GV-based artificial cells containing DNA (L 3200, M 1164, and S 374 bp). The increase ratios of GVs were measured by confocal scanning laser microscopy. For GV(L) vs. GV(M): GV(L) membrane was tagged with BODIPY-HPC, and GV(M) membrane was tagged with Texas Red-DHPE (**a**), and for GV(M) vs. GV(S): GV(M) membrane was tagged with BODIPY-HPC, and GV(S) membrane was tagged with Texas Red-DHPE (**b**).

The molar ratio of membrane lipids in this experiment was POPC, **V**, **C**, and cholesterol and PEG-grafted phospholipid (DSPE-PEG1000,) = 78:4.2:8.5:8.5:0.85 mol%), in accordance with a previous experiment [6]. The ratio between the total membrane lipids and precursor of membrane lipids V* is 1 to 1, which means that a dispersion contains enough V* to double the number of GVs. To investigate the difference in the increase ratios of GVs in the competitive proliferation between two different kinds of GV-based artificial cells [GV(L; 3200 bp), GV(M; 1164 bp), and GV(S; 374 bp)] by confocal laser scanning fluorescence microscopy, we added the membrane lipid precursor V* to a mixture of dispersions of GV(L) and GV(M) or a mixture of dispersions of GV(M) and GV(S) (Figure 4). If no intervention occurred between two kinds of GVs, the increase ratio of GVs was regarded as an average of the noncompetitive ratios of the two kinds of GVs and estimated to be 133% and 100% for GV(M)-GV(L) and GV(M)-GV(S), respectively (Table 1). However, the actual increase ratio as a whole was 105% in the case of GV(L)-GV(M) and 72% for GV(M)-GV(S) (Table 2 and Table 3). Hence, we confirmed that the predominance of GV(M) over GV(L), which was observed in noncompetitive proliferation, was amplified in competitive proliferation (Table 4, third column from left above). On the other hand, when GV(M) and GV(S) were mixed and competed, the predominance of GV(M) disappeared unexpectedly, and the difference in the ratios between GV(M) and GV(S) decreased appreciably (Table 4, third column from left below).

Noncompetitive increase ratios of GV(L), GV(M) and GV(S) were calculated independently. Measurement of the increase ratio of GVs was conducted at 48 h after PCR and at 45 min after the addition of V*. In the noncompetitive proliferation between GV(L) and GV(M), a significant difference (*p* = 0.502) was not detected. In the combination between GV(M) and GV(S), the increase ratios of these two in the noncompetitive proliferation showed a significant difference at the 2% significance level (*p* = 0.018). The significant differences were revealed by the Wilcoxon rank-sum test with the Bonferroni correction. Each number of samples was five for each condition.

Competitive increase ratios of GV(L) and GV(M) were calculated independently. Measurement of the increase ratio of GVs was conducted at 48 h after PCR and at 45 min after the addition of V*. A significant difference was established at the 1% significance level (*p* = 0.009) in the competitive proliferation between GV(L) and GV(M) by the Wilcoxon rank-sum test, regardless of the significant difference was not detected in the noncompetitive condition. Each number of samples was five for each condition.

Competitive increase ratios of GV(M) and GV(S) were calculated independently. Measurement of the increase ratio of GVs was conducted at 48 h after PCR and at 45 min after the addition of V*. A significant difference was not obtained (*p* = 0.754) in the competitive proliferation between GV(L) and GV(M) by the Wilcoxon rank-sum test, regardless of the significant difference was detected in the noncompetitive condition. Each number of samples was five for each condition.

The rate of increase ratios of GV(L) or GV(S) to GV(M) were calculated using increase ratios under the noncompetitive and competitive conditions in Table 1 to demonstrate the interaction between GVs.

It should be noted that experiments on the competitive proliferation of GV-based artificial cells containing different chain length DNA (M; 1164 bp, S; 374 bp) were carried out using a more sensitive confocal scanning laser microscope. The reason for re-examining the above experiments was that the results obtained from the measurement described in the text were rather unexpected. To explore the reason for this unpredictability, a more precise experiment was required. The result obtained by a confocal microscope with higher sensitivity was consistent with the result described in the text (Figure S8 and Table S2).

#### 3.2.2. Flow Cytometry Measurement of Competitive Proliferation

**Figure 5:**
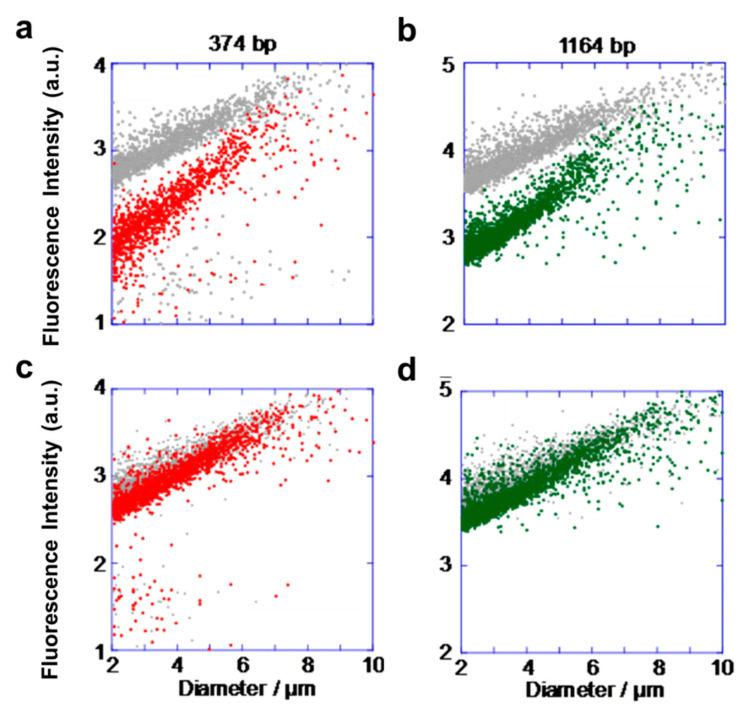
Flow cytometry diagrams showing the correlation between the diameters which is given by correlation with size of polystyrene beads (Figure S9) and fluorescence intensities of GV(S) and GV(M) in competitive proliferation. The two diagrams in the left column (**a)** and (**c**) correspond to population changes of GV(S). The two diagrams in the right column (**b**) and (**d**) correspond to population changes of GV(M). The ratio of V* and total membrane lipids is 3:1 in the top row and 1:3 in the bottom row.

The ratios of the increased number of GV(M) and GV(S) were also measured by flow cytometry to examine the reason why the difference in the increase ratios between GV(M) and GV(S) decreased. The population diagrams of GV(M) and GV(S) in the competitive condition were measured by flow cytometry (Figure 5). The diameter of a vesicle was estimated from a calibration curve indicating the correlation with a pulse width of the forward scattered light intensity using polystyrene beads with fixed sizes (Figure S9). The correlation between the diameters of GVs and the fluorescence intensities emitted from the GV membrane stained with a fluorescent probe exhibited a cluster of dots extended in a diagonal direction; GV(S) was stained with Texas Red-DHPE, and GV(M) was stained with BODIPY-HPC. The two diagrams shown in the left column in Figure 5 indicate the time dependence of populations of GV(S) immediately after the addition of V* (gray dots in the top plot) and at 2 h after the addition (red dots in the bottom plot). The ratio of added V* (precursor of membrane lipid **V**) to the total lipids was 3:1 in the top diagram and 1:3 in the bottom diagram. The shift of dot plots from the original plots at 2 h after the addition of V*(1:1) was intermediate between the shifts observed in these two diagrams (Figure S10).

Based on the analysis of the microscope images and the flow cytometry data, the interaction between proliferation dynamics is interpreted as follows. In the case of GV(L) and GV(M), both kinds of GVs exhibit budding deformation. However, since the deformation of GV(L) proceeds more slowly than that of GV(M), the precursor is predominantly taken up by GV(M) under competitive conditions. As a result, the rate of increase ratio of GV(M) to GV(L) becomes larger than the rate under noncompetitive conditions. On the other hand, in the case of GV(M) and GV(S), the competitive proliferation proceeded almost equally despite the distinct predominance of GV(M) over GV(S), as observed in the noncompetitive proliferation (Table 2). This discrepancy may be explained on the basis of the data obtained by flow cytometry during noncompetitive proliferation [6]. According to the decrease in fluorescence intensity in the noncompetitive experiment, the division of GV(S) occurred quickly and produced smaller GVs than those from GV(M). Immediately after the addition of V*, some GV(S) divided into many small GVs (GVs with diameters lower than 3 μm were not counted as artificial cells from the viewpoint of viability). This means that the amount of added V* decreased rapidly, accompanied by rapid division. Since the amount of V* added to the dispersion of GV-based artificial cells was the same (the ratio of V* and total membrane lipids was 1:1) in both noncompetitive and competitive proliferations, the remaining amount of V* had to be shared with two kinds of GVs (1164 and 374 bp) in competitive proliferation, which substantially suppressed the division of GV(M). This influence must be the main factor of the unexpected averaging effect on the increase ratio of GV(M) and GV(S). Another factor was a leveling-off effect on the size of divided GVs, in particular, in the case of GV(S) induced by dilution of the V* concentration. As seen from our precedent examples, a GV containing shorter DNA is likely to produce smaller GVs when a high concentration of V* is added. This tendency was reduced in a dilution of V*. Hence, the frequency of producing daughter GV(S) satisfying the viability condition for an artificial cell (not less than 5 μm) would be increased.

To confirm this interpretation, we conducted flow cytometry measurements focusing on the difference in the increase ratio of GV(M) and GV(S). Although the rate of increase ratios of GV(M) to GV(S) was 2.4 in the noncompetitive experiment, it changed to 1.1 under competitive conditions (Table 4). This value, however, was restored when the amount of added V* increased. In practice, we increased the molar concentration ratio of V* to total membrane lipids from 1:3, 1:1 to 3:1 to see the effect (Table 5). The observed tendency was consistent with the interpretation discussed above.

The left column represents the relative concentrations of V*, and the right column represents the rate of increase ratio GVs [GV(M)/GV(S)]. Errors are calculated from the confidence interval of 99% on the basis of the normal distribution.

It can be safely said that a dominant species among the GV-based artificial cells, GV(M), appeared in competitive proliferation. However, it turned out that the concentration of nutrients (V* in the current experiment) influenced the appearance of the dominant species. There is a chance for a fast-eating species to become a rival for the predominant species in the competitive condition because the competition for obtaining nutrients is more severe [15].

## 4. Discussion

### 4.1. Discussion on Presented Results

“Phenotypic plasticity and competitive proliferation” as described in the text, our GV-based artificial cell is endowed with a primitive information flow started from DNA of a defined chain length which is intruded in the GV membrane and forms a complex (**C**@DNA) with gathered cationic catalyst **C**, serving as a lipo-deoxyribozyme which regulates the proliferation dynamics. The informational system is much flexible compared with a contemporary transcription and translation system regulated by the sequence of base pairs of DNA. The flexibility was rationalized by the result obtained from two major experiments on the phenotypic plasticity and competitive proliferation, influenced by the external environments, one was the nutrition poor condition and the other was the existence of other species of artificial cell containing DNA with different chain length.

### 4.2. Discussion on Smart Artificial Cells

It is worth noting that the primitive information flow in the artificial cell differentiates itself from molecular machines and robots [16] so far developed, as the artificial cells have shown that the length of DNA can serve as information [17]. Of course, if a molecular robot carries a determination device like a microcomputer, it can select a suitable response based on installed information towards the external condition. However, it would be hardly possible to produce different phenotypes in response to the external condition. We suggest that, in addition to the above-mentioned features, our artificial cell acquired the smart functions required for an intelligent artificial cell robot. They are, for example, repetitive proliferation [18], and movement in response to external stimuli such as light [19], surfactant [20], pH changes [21], etc.

**Figure 6:**
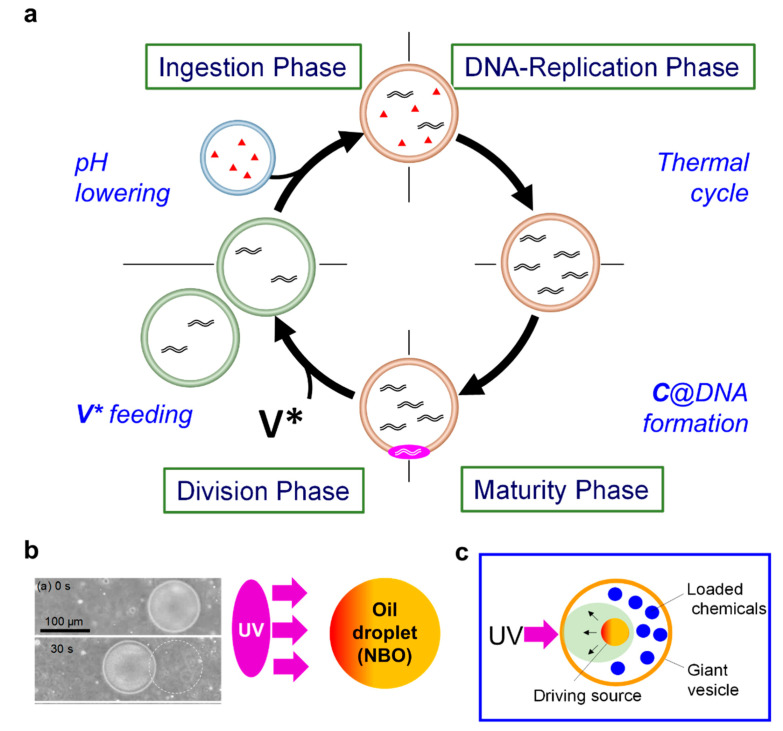
Phototactic movement of an oil droplet (**a**), a GV-based artificial cell (**b**), and a recursive GV-based artificial cell with four cellular phases (**c**).

“Recursive proliferation” of the GV-based artificial cell requires a solution of lack of the levels of dNTP, DNA polymerase, and cationic catalyst **C** in the GV through sequential growth and division. In addition, the surface charge of the GV should be regulated to not trend towards being too cationic in order to facilitate cationic V* uptake [22]. We resolved these problems by intervesicular fusion with a conveyer GV with the opposite surface charge, triggered by the lowered pH. Both depleted substrates in the GV and the acidic surface charge of the GV were restored by vesicular fusion with an anionic (POPG-rich) conveyer GV that carries a sufficient number of substrates [23,24]. Using this method, a GV-based artificial cell acquired recursiveness, and proliferation was able to proceed repeatedly, allowing cycling of a primitive cell cycle with four phases (Figure 6a) [13]. The intervesicular delivery of substrates corresponds to endocytosis of a living cell. The characteristic point of this recursive artificial cell is that external and internal stimuli drive the primitive cell cycle; the thermal cycle promotes DNA replication, the intrusion of amplified DNA into the membrane promotes maturation of the catalytic activity, the feeding of V* promotes cellular division, and the lowered pH promotes fusion of the artificial cell with a transporter GV for nutrient ingestion. In contrast to Stoddart’s molecular shuttle which moves randomly by thermal energy based on the classical thermodynamics [25], the primitive cell cycle turns in a clockwise manner as a supramolecular ratchet, responding to these series of stimuli on the basis of high dynamic hierarchy. This artificial cellular robot exhibits the ability of generation turnover.

As for “Ingredients release by photochemical reactions” from an artificial cell, we prepared a photo-pierceable GV [26]. Upon UV irradiation of such a GV composed of phospholipids and photo-responsive amphiphiles, the release of a chemical, e.g., water-soluble fluorescence dye, to the exterior water phase was confirmed by fluorescence microscopy [15]. An emptied GV maintained its spherical shape even after release due to the strong surface tension of the GV membrane [16]. This GV-based artificial cell can operate in on-site medical care as a molecular robot [27].

“Self-propulsion induced by photochemical reactions” is very important to drive a cell towards a nutrient-rich environment. The chemotactic self-propulsion of an oil droplet comprising oleic anhydride has been reported [28]. The unique behavior is expected to afford a new concept for designing microrobotics [19,29]. An emulsion comprising of oil droplets of a caged oleic acid, 2-nitrobenzyl oleate (NBO), exhibited unidirectional movement towards a UV light source (Figure 6b) [30]. Namely, it shows positive photo-controllable behavior. The surface tension of the droplet’s photo-irradiated surface (front side of the droplet) becomes lower than that at the rear side. Hence, Marangoni flow emerged from the front side to the rear of the droplet at the boundary between the oil and water phases, and the water flow drives the unidirectional movement of the droplet towards a UV light source. If such oil droplets are encapsulated in GV-based protocell, it may have a chance to exhibit macroscopic movement by photo-irradiation. An investigation along this line is in progress (Figure 6c).

The recursiveness of the proliferation of an artificial cell is crucial to consider the evolution of the artificial cell [31]. The processes applied to the recursive artificial cell can also be applied to mingle different kinds of DNA in GVs with each other. If competitive proliferation is conducted using DNA-containing, GV-based artificial cells, fusion and division should afford artificial cells containing a mingled DNA composition, and a cell containing a certain composition may become a dominant species, responding to the external environment. This process means the emergence of evolved species [32].

Evolution should be located at the highest stage of the hierarchical dynamics in self-proliferation, and it should provide the adaptable species under the given environment, spontaneously. This concept may open a new horizon, “evolution”, for intelligent molecular machines and robotics [33].

## Figures and Tables

**Table 1:** Increase ratios of GV(L), GV(M), and GV(S) under noncompetitive condition.

GV	Noncompetitive Increase Ratio
GV(L)	125 ± 13 %
GV(M)	141 ± 14 %
GV(S)	59 ± 9 %

**Table 2:** Increase ratios of GV(L) and GV(M) under competitive condition.

GV	Competitive Increase Ratio
GV(L)	76 ± 7 %
GV(M)	133 ± 7 %

**Table 3:** Increase ratios of GV(M) and GV(S) under competitive condition.

GV	Competitive Increase Ratio
GV(M)	75 ± 8 %
GV(S)	69 ± 5 %

**Table 4:** Rates of increase ratios of GV(M) to GV(L) and of GV(M) to GV(S) in competitions.

Rate of Increase Ratio	Noncompetition	Competition
GVM/GV(L)	1.1	1.8
GV(M)/GV(S)	2.4	1.1

**Table 5:** Dependence of the relative concentration of V* on the rate of increase ratios of GV-based artificial cells.

Rate [Total Lipid ]/[V*]	Rate of Increase Ratio
3/1	1.1 ± 0.10
1/1	1.2 ± 0.11
1/3	1.8 ± 0.20

## Supplementary Materials

The following are available online at https://www.mdpi.com/2072-666X/11/6/606/s1, Figure S1: Dependence of FRET intensity on starvation period. Figure S2: Dependence of relative yield of MT on **V*** concentration. Figure S3: Influence of distribution of **C**@DNA in the membrane on the morphological patterns. Figure S4.: Confocal microscopy images of GV fused between pre-BD with MT at 6 h after mixing. Figure S5: Confocal microscopy image of a mixed dispersion of BD and pre-BD at 6 h after mixing. Figure S6: Fluorescence intensity distribution of a mixed dispersion of pre-MT stained with BODIPY-HPC and pre-BD stained with Texas Red-DHPE. Figure S7: Dependence of fluorescence distributions of MT and pre-BD on the mixed ratios of those two GVs in the dispersion. Figure S8: Confocal fluorescence microscopy images of GVs before, at 2 h and at 24 h after the addition of **V***. Figure S9: Correlation between bead’s diameter and pulse width of forward scatter. Figure S10: The population diagrams of GV(S) (a) and GV(M) (b) in the competitive condition. Table S1: Membrane compositions of GVs and induced morphological patterns after starvation period of 3 h. Table S2: Competitive proliferation between GV (S) stained with Texas-Red DHPE and GV (M) stained with BODIPY-HPC. Table S3: Competitive proliferation between GV (S) stained with BODIPY-HPC and GV (M) stained with Texas-Red DHPE. Table S4: Dependence of GV numbers on concentration ratios of total lipid and **V***. Note S1: Mechanism of starvation period-dependent morphological change. Video S1: Multiple tubulation.
