# Supplementary material for: Environment-Sensitive Intelligent Self-Reproducing Artificial Cell with a Modification-Active Lipo-Deoxyribozyme
Source: Micromachines (Basel). 2020 Jun 22;11(6):606. doi: 10.3390/mi11060606 (PMC7344958; doi:10.3390/mi11060606)
Supplement: Supplementary file 1 [file micromachines-11-00606-s001.zip › Supplementary files/Supplementary Information.pdf]

Supplementary Information

Environment-sensitive Intelligent Self-reproducing Artificial Cell with Modification-active Lipo-deoxyribozyme

Muneyuki Matsuo, Yuiko Hirata, Kensuke Kurihara, Taro Toyota, Toru Miura, Kentaro Suzuki, and Tadashi Sugawara*

Supplementary Figures

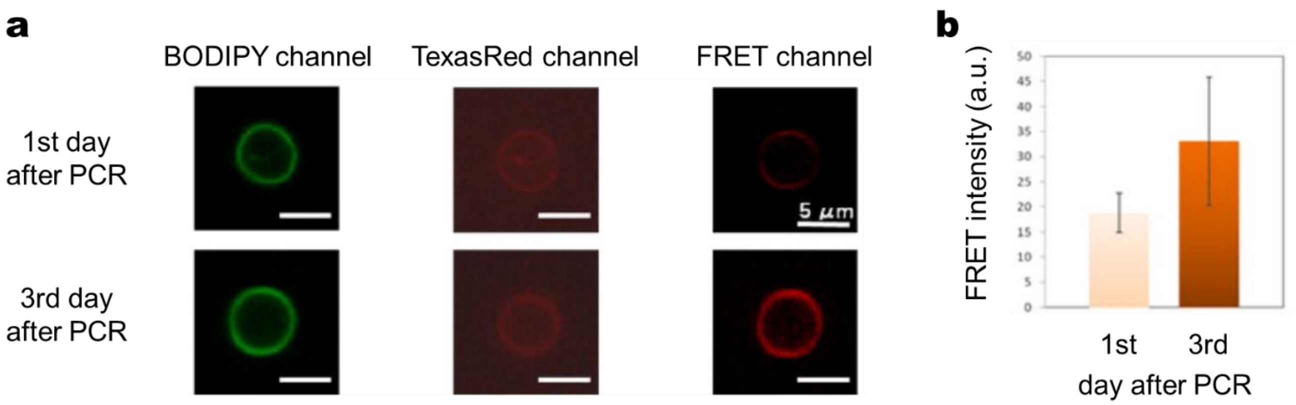

Figure S1. Dependence of FRET intensity on starvation period. Fluorescence intensities derived from Förster excited energy transfer (FRET) upon the access of catalyst **C**-tagged with BODIPY and DNA-tagged with Texas Red, both of which constitute **C**@DNA, was measured. (a) Confocal fluorescence microscope images measured on first and third days after PCR performance. Scale bars represent 5 μm. (b) Average values of highest FRET intensities of GVs on first (n = 5) and third days (n =3) after PCR performance were treated by the Wilcoxon rank-sum test to reveal the difference at the 5% significance level (p = 0.025). The highest FRET intensity was evaluated from the averages of the maximum intensities of 8 line profiles crossing over a GV. Error bars represent S.D.

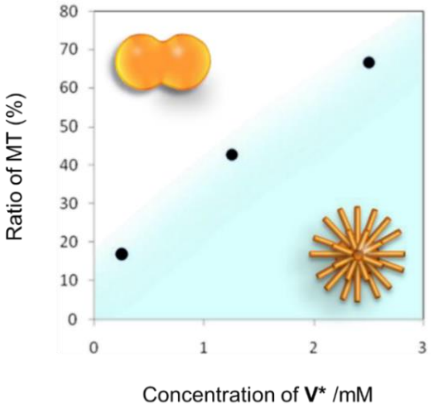

Figure S2. Dependence of relative yield of MT on V* concentration. Self-reproduction dynamics was observed by differential interference microscope during 30 min after addition of **V*** (0.63 mM, 1.3 mM, 2.5 mM). Formation ratios of **MT** to all deformed GVs were plotted.

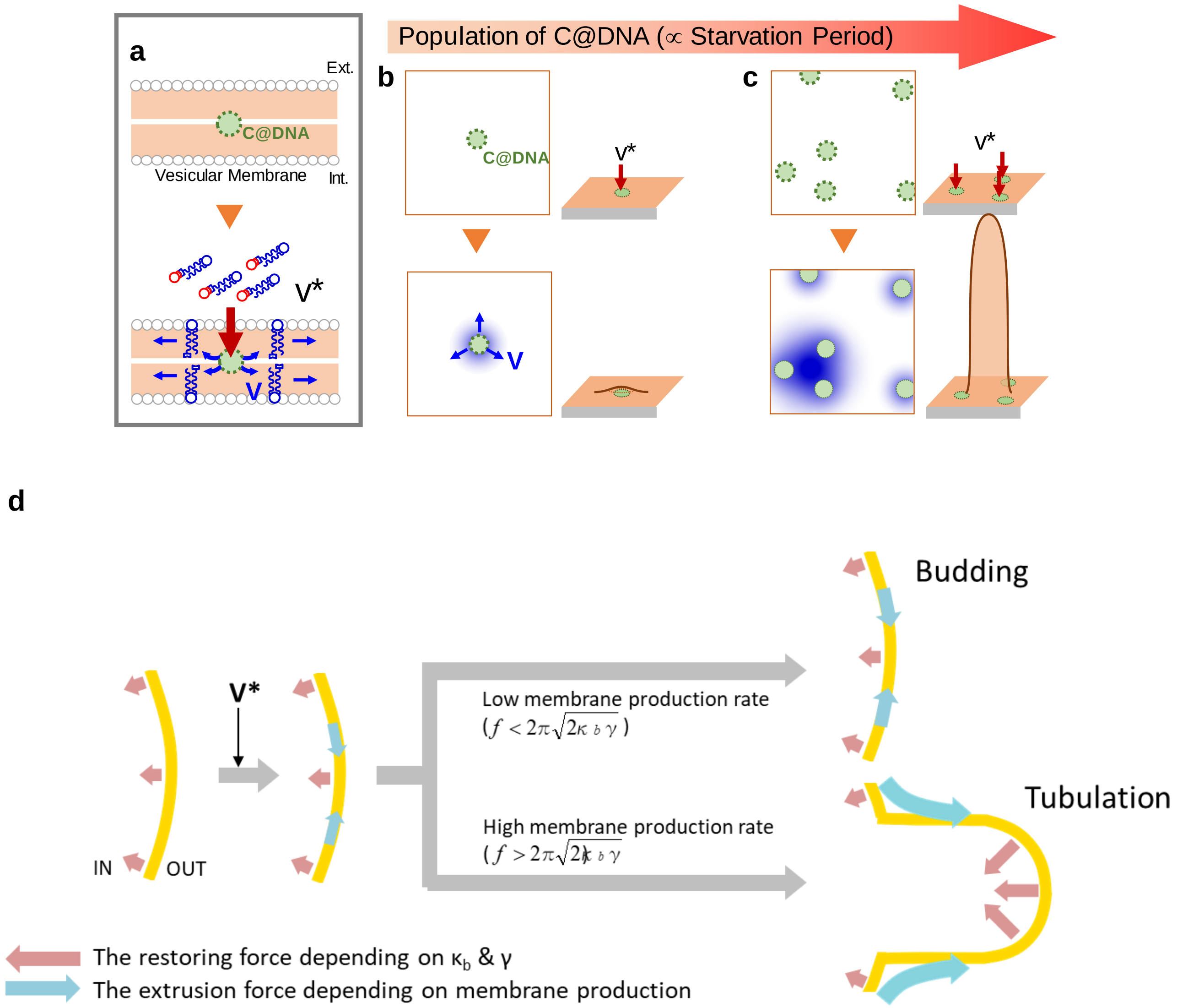

Figure S3. Influence of distribution of C@DNA in the membrane on the morphological patterns. (a) Formation and diffusion of membrane lipid **V** from its precursor **V*** in the vicinity of **C**@DNA in vesicular membrane. (b) Budding deformation around **C**@DNA at low surface density of **C**@DNA, induced by the addition of **V***. Produced membrane lipid **V** diffuses isotopically from **C**@DNA. (c) As the surface concentration of **C**@DNA increases, high compressibility caused by generated **V** in a zone surrounded by **C**@DNAs induces tubular deformation in the middle of the zone. (d) At low surface density of **C**@DNA, the force generated by membrane production is lower than a certain threshold and a GV is budded. At high density of **C**@DNA, the force f is at the threshold or higher, and a GV generates a tube. κ_b and γ represent bending rigidity and surface tension, respectively. The effect of **C**@DNA distribution and the estimation of the threshold were explained in Note S1.

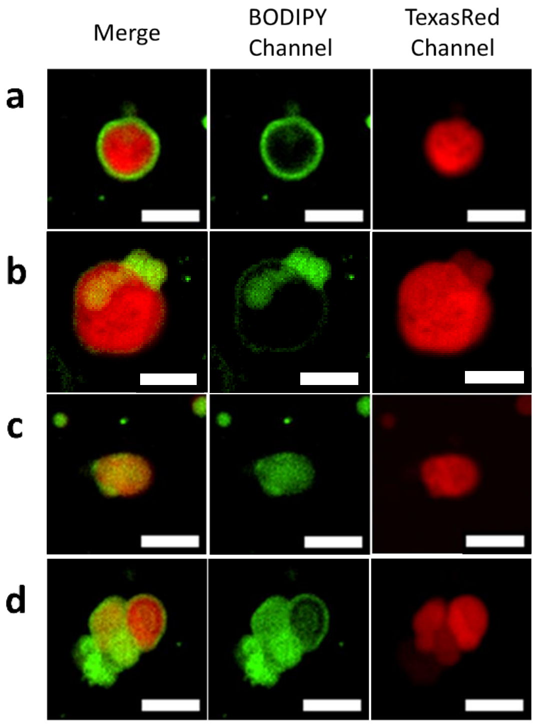

Figure S4. Confocal microscopy images of GV fused between pre-BD with MT at 6 h after mixing. Merge channel (left), BODIPY channel (center) and Texas Red channel(right). Scale bar represents 10 μm. Images showed following four patterns; a. spherical pre-BD coated with MT, b. pre-BD partially coated with MT, c. pre-BD merged with MT, d. clustered BD coated and connected with MT.

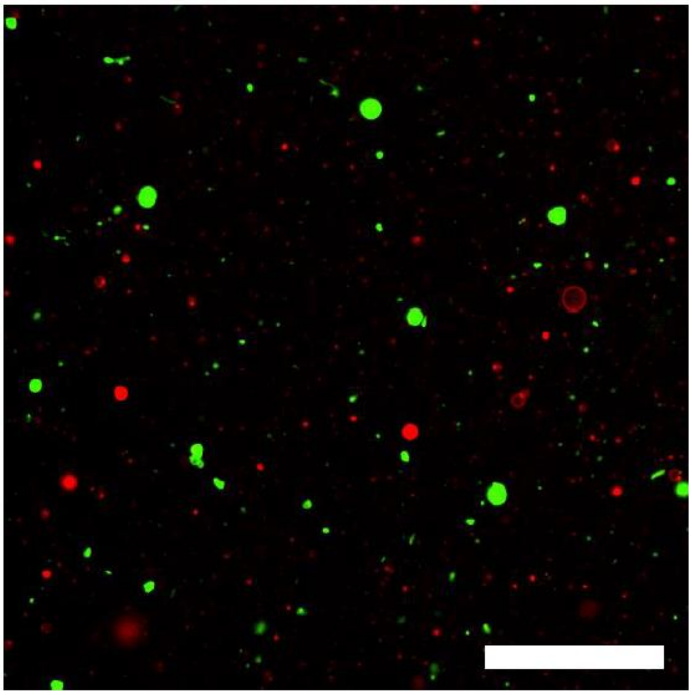

Figure S5. Confocal microscopy image of a mixed dispersion of BD and pre-BD at 6 h after mixing. BD and pre-BD were stained with BODIPY-HPC and Texas Red-DHPE, respectively. Scale bar represents 50 um.

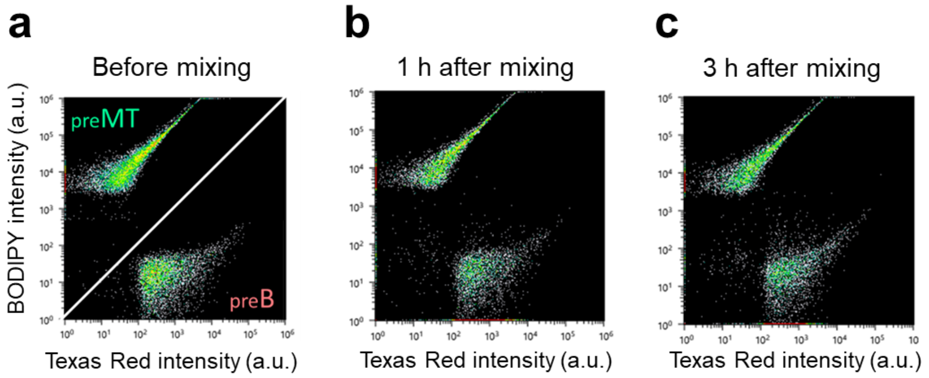

Figure S6. Fluorescence intensity distribution of a mixed dispersion of pre-MT stained with BODIPY-HPC and pre-BD stained with Texas Red-DHPE. (a) before addition, (b) at 1 h after mixing two dispersions, (c) at 2h after mixing two dispersions. Vertical axis represents the intensity of BODIPY-HPC (green) and horizontal axis represents the intensity of Texas Red-DHPE (red).

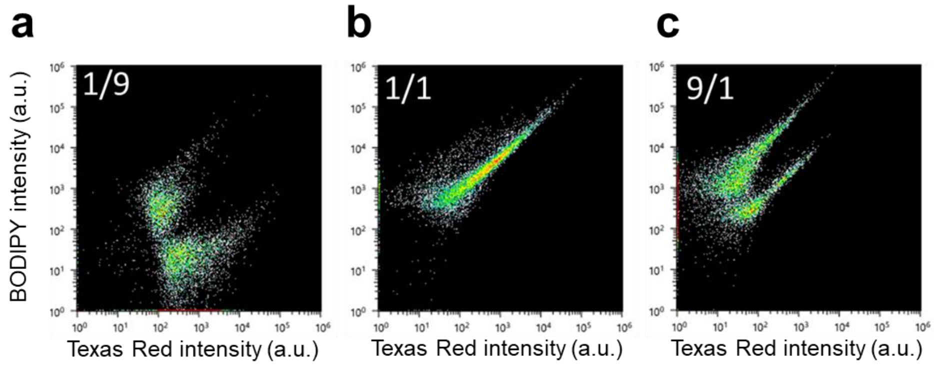

Figure S7. Dependence of fluorescence distributions of MT and pre-BD on the mixed ratios of those two GVs in the dispersion. MT was stained with BODIPT-HPC, and pre-**BD** was with Texas Red-DHPE (a) Mixed ratio between **MT** and pre-**BD** was 1:9, (b) **MT** and pre-**BD** was 1:1, (c) **MT** and pre-**BD** was 9:1. The variance-in the fluorescence intensities of BODIPT-HPC against Texas Red-DHPE of the 1:9 mixture was larger than those obtained from the samples with the concentration ratios of 1:1 and 1:9. All vesicular dispersions were incubated for 72 h after PCR, and microscope observation was conducted at 2 h and 24 h after addition of **V***. The result suggests that pre-**BD** was coated by plural tubes of **MT**s but there was an upper limitation of the number of coating tubes of **MT**s.

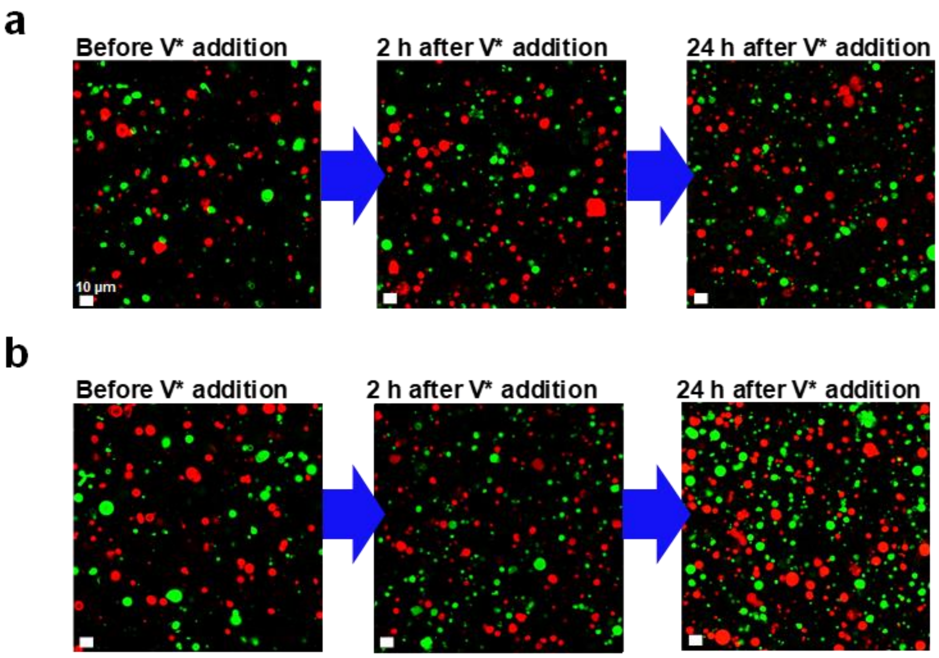

Figure S8. Confocal fluorescence microscopy images of GVs before, at 2 h and at 24 h after the addition of V*. GV(S) was tagged with Texas Red-DHPE and GV(M) was tagged with BODIPY-HPC (a). GV(S) was tagged with BODIP-HPC and GV(M) was tagged with Texas Red-DHPE (b). Compositional ratios of Texas Red-DHPE and BODIPY-HPC were 0.05 mol% and 0.1 mol%, respectively. Concentrations of total lipid and **V*** solution were the same as 1.3 mM. After PCR, a vesicular dispersion was incubated for 72 h, microscope observation was conducted at 2 h and 24 h after addition of **V*.** Scale bars represent 10 μm. Analyzed data from these images was shown in tables S2 and S3.

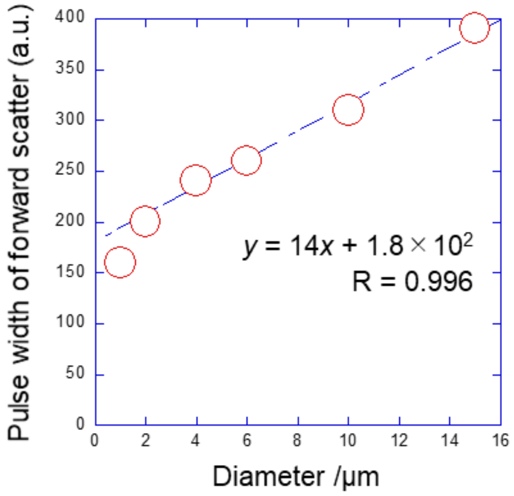

Figure S9. Correlation between bead's diameter and pulse width of forward scatter. Similar to the previous investigation [1], a calibration curve was obtained by shedding a dispersion of beads with a diameter of 0.99, 1.9, 3.7, 5.6, 9.9 and 15 μm and measuring pulse widths of forward scatter light intensities by a flow cytometer because we were interested in GVs larger than 2 μm in the current investigation.

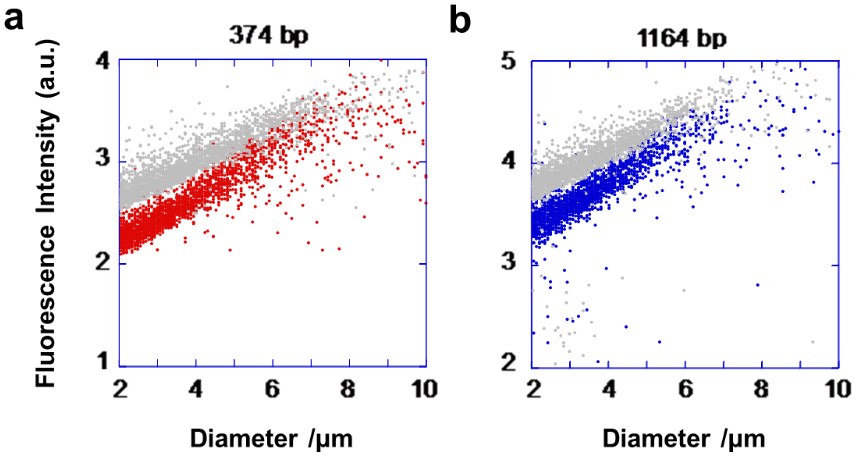

Figure S10. The population diagrams of GV(S) (a) and GV(M) (b) in the competitive condition. The ratio of **V*** and total membrane lipids was 1:1.

Supplementary Tables

Table S1. Membrane compositions of GVs and induced morphological patterns after starvation period of 3 h.

No	Membrane Composition (mol%)			Excess Negative Charge (%)	Deformation Patterns (%)	
	POPC (+,-)	POPG (-)	**V** (+)		Budding	Multiple tubulation
1	50	**18**	**18**	-	100 (4/4)	0.0 (0/4)
2	50	**21**	**15**	2	100 (9/9)	0.0 (0/9)
3	50	**24**	**12**	8	83 ± 6.2 (5/6)	17 ± 6.2 (1/6)
4	**42**	**32**	12	16	88 ± 4.1 (7/8)	13 ± 4.1 (1/8)
5	**35**	**39**	12	23	86 ± 2.5 (12/14)	14 ± 2.5 (2/14)

The ratios of amphiphilic catalyst **C** and cholesterol were 9.0 mol% and 5.0 mol% for all membrane compositions, respectively. Excess negative charge was calculated as [POPG % - (**V** % + (**C** %/2))]. The cationic catalyst **C** contains an imidazolium hydrochloride. Because the acidity of the imidazolium hydrochloride is weak, about a half of catalyst are deprotonated (pKa ≈ 8). Hence cationic **C** was divided by two in the process of calculation under the current buffer condition (pH = ca.8). Errors in the probability of the morphological patterns are calculated as a standard error (SE = $\sigma \cdot n^{-1/2}$, σ: standard deviation) corresponding to individual numbers of samples from their populations.

Table S2. Competitive proliferation between GV (S) stained with Texas-Red DHPE and GV (M) stained with BODIPY-HPC.

Time /h	Number of GV(S)	Number of GV(M)	Ratio [GV(M)/GV(S)]
0 h (before addition of V*)	27±11	26±7	0.96
2 h	48±22	36±14	0.75
24 h	55±27	49±25	0.89

Table S3. Competitive proliferation between GV (S) stained with BODIPY-HPC and GV (M) stained with Texas-Red DHPE.

Time /h	Number of GV(S)	Number of GV(M)	Ratio [GV(M)/GV(S)]
0 h (before addition of V*)	51±10	79±9	1.5
2 h	67±17	86±12	1.3
24 h	82±13	92±29	1.1

Table S4. Dependence of GV numbers on concentration ratios of total lipid and V*.

Total lipid : V*		0 min	10 min	20 min	30 min	60 min	120 min
1:9		Unable to analyze					
1:3	1164GV	1477	1510	1428	1401	1445	1339
	374GV	1351	843	720	724	786	738
	Ratio	1.09	1.79	1.98	1.93	1.84	1.81
1:1	1164GV	1516	1270	1269	1208	1287	1363
	374GV	1274	1084	1100	1019	1111	1179
	Ratio	1.19	1.17	1.15	1.18	1.15	1.15
1:(1/3)	1164GV	1481	1481	1521	1561	1529	1531
	374GV	1372	1355	1317	1312	1386	1384
	Ratio	1.08	1.09	1.15	1.19	1.10	1.10
1:(1/9)	1164GV	1651	1747	1642	1709	1719	1702
	374GV	1506	1469	1504	1506	1535	1540
	Ratio	1.10	1.19	1.09	1.13	1.12	1.11

GVs the diameter of which is equal to or not less than 2 μm were listed in reference to the ratio of concentrations of total membrane lipids to **V*** (membrane lipid precursor).

Supplementary Note

Note S1. Mechanism of starvation period-dependent morphological change.

A crucial influence of the distribution of **C**@DNA, which is increasingly embedded in the vesicular membrane depending of the starvation period, on the pattern of morphological changes of the GV-based model protocell (Figure S3a). One of the interpretations on this phenomenon is described below. The morphological change of the GV-base model protocell in the current case is classified into two phases. In the former phase, the ordinary deformation occurs predominantly. After a short starvation period, the surface concentration of **C**@DNA in the vesicular membrane is low with relatively long center to center distances between **C**@DNAs. As a result, the lateral compressibility caused by the produced membrane lipids **V** in a surrounding zone around **C**@DNA is not high enough to induce the budding (Figure S3b).

After a long starvation period, the lateral compressibility caused by accumulated **V** within the zones surrounded by many **C**@DNAs becomes sufficiently high. At the same time, the diffusional escape of **V** from the zone is suppressed by surrounding **C**@DNAs as a barrier for the diffusional escape. A high lateral compressibility in the zone causes a buckling of the membrane to generate the **MT** as a kinetically controlled structure. To confirm this scenario, we examined the dependence of **MT** ratio on the concentration of the added **V*** under the same condition of DIC microscopy observation. As the concentration of **V*** became higher, the ratio of the multiple tubulation increased due to the increase in the formation rate of **V** even though the amount of **C** is the same (Figure S3c).

If forece derived from matured **C**@DNA acquires the potentiality to exceed the threshold value defined below, such **C**@DNA is able to convert **V*** to **V** rapidly all over the GV membane, and transforms itself into a multi-tublated GV (Figure S3d). In the Helfrich's model, membrane free energy of tubulated vesicle with stress f is written as (κ_b: bending rigidity, H: mean curvature, A: area, ΔP: Pin-Pout, V: volume, L: length of tube) [2]

$$G_{tot} = 2\kappa_b \int H^2 dA + \frac{1}{2}\kappa_a \left(\frac{\Delta A}{A_0}\right)^2 - \Delta PV - fL$$

If the tubulation is a quasi-static process, tubulation to outer side becomes thermodynamically favorable with f satisfying the following inequality, taking $(\partial G_{tot}/\partial R_{sp})$, $(\partial G_{tot}/\partial R_{cy})$, $(\partial G_{tot}/\partial L) < 0$ (R_{sp}: sphere radius, R_{cy}: tube radius, γ: surface tension) [3]

$$f > 2\pi\sqrt{2\kappa_b \gamma}$$

If f becomes larger than the right term, GV is able to grow tubules from the membrane surface.

Supplementary References

1. M. Matsuo, *et al.*, *Sci. Rep.*, **9**, 1-11 (2019).

2. W. Helfrich, *et al.*, *J. Phys.*, **37**, 1335 (1976).

3. D. J. Bukman, *et al.*, *Phys. Rev. E*, **54**, 5463 (1996).
